# Airway Surface Dehydration Aggravates Cigarette Smoke-Induced Hallmarks of COPD in Mice

**DOI:** 10.1371/journal.pone.0129897

**Published:** 2015-06-12

**Authors:** Leen J. M. Seys, Fien M. Verhamme, Lisa L. Dupont, Elke Desauter, Julia Duerr, Ayca Seyhan Agircan, Griet Conickx, Guy F. Joos, Guy G. Brusselle, Marcus A. Mall, Ken R. Bracke

**Affiliations:** 1 Laboratory for Translational Research in Obstructive Pulmonary Diseases, Department of Respiratory Medicine, Ghent University Hospital, Ghent, Belgium; 2 Department of Translational Pulmonology, Translational Lung Research Center Heidelberg (TLRC), Member of the German Center for Lung Research (DZL), University of Heidelberg, Heidelberg, Germany; University Hospital Freiburg, GERMANY

## Abstract

**Introduction:**

Airway surface dehydration, caused by an imbalance between secretion and absorption of ions and fluid across the epithelium and/or increased epithelial mucin secretion, impairs mucociliary clearance. Recent evidence suggests that this mechanism may be implicated in chronic obstructive pulmonary disease (COPD). However, the role of airway surface dehydration in the pathogenesis of cigarette smoke (CS)-induced COPD remains unknown.

**Objective:**

We aimed to investigate *in vivo* the effect of airway surface dehydration on several CS-induced hallmarks of COPD in mice with airway-specific overexpression of the β-subunit of the epithelial Na^+^ channel (βENaC).

**Methods:**

βENaC-Tg mice and wild-type (WT) littermates were exposed to air or CS for 4 or 8 weeks. Pathological hallmarks of COPD, including goblet cell metaplasia, mucin expression, pulmonary inflammation, lymphoid follicles, emphysema and airway wall remodelling were determined and lung function was measured.

**Results:**

Airway surface dehydration in βENaC-Tg mice aggravated CS-induced airway inflammation, mucin expression and destruction of alveolar walls and accelerated the formation of pulmonary lymphoid follicles. Moreover, lung function measurements demonstrated an increased compliance and total lung capacity and a lower resistance and hysteresis in βENaC-Tg mice, compared to WT mice. CS exposure further altered lung function measurements.

**Conclusions:**

We conclude that airway surface dehydration is a risk factor that aggravates CS-induced hallmarks of COPD.

## Introduction

Efficient mucociliary clearance is an essential innate defence mechanism of the lung [1–4]. Although ciliary activity and mucus secretion play an important role in airway mucus clearance, evidence from biophysical studies indicates that the hydration state of the airway surface is the key determinant [5, 6]. While airway surface dehydration is a well-established disease mechanism in cystic fibrosis [5, 7], recent research suggests that this abnormality may also play a role in chronic obstructive pulmonary disease (COPD) [8–11]. Pathologically, COPD is mainly caused by cigarette smoking and characterized by mucus obstruction of the small airways [12], chronic pulmonary inflammation, obstructive bronchiolitis and emphysema [13, 14].

Several studies demonstrated that cigarette smoke (CS) has detrimental effects on the hydration of airway surfaces. First, it was shown that CS affects ion channels in the apical membrane of airway epithelial cells, thereby disturbing the balance between Na^+^ absorption and Cl^-^ secretion and leading to airway surface dehydration. Most notably, CS induces an acquired deficiency of the cystic fibrosis transmembrane conductance regulator (CFTR), a crucial cAMP-dependent Cl^-^ channel that is mutated in cystic fibrosis [8–10]. In chronic smokers, CFTR function is reduced to ~ 45% of normal and mucus is hyperconcentrated *in vivo* [8, 9]. This acquired CFTR dysfunction contributes to inadequate mucociliary transport [10] and is associated with chronic bronchitis and dyspnoea in smokers with and without COPD [15]. Furthermore, exposure to CS extract enhances the activity of the epithelial Na^+^ channel (ENaC) in alveolar type I and type II cells [16], suggesting that CS exposure results in a hyposecretory/hyperabsorptive ion transport phenotype. Along these lines, recent studies on the protein levels of CFTR and ENaC in lung tissue of COPD patients demonstrated that CFTR levels were positively correlated with lung function, whereas levels of α- and βENaC showed a negative correlation with lung function [17]. In mice, an imbalance between Na^+^ absorption and Cl^-^ secretion, has been achieved in βENaC transgenic (βENaC-Tg) mice. In these mice, airway-specific overexpression of βENaC causes constitutive airway surface dehydration and spontaneous chronic obstructive lung disease characterized by airway mucus obstruction, neutrophilic inflammation and development of emphysema early in life [7, 18].

A second mechanism by which CS can contribute to airway surface dehydration, is the CS-induced mucin hypersecretion [3, 11, 13]. The two major secreted mucins in airways, MUC5AC and MUC5B, are both increased in patients with COPD [19–21]. These mucin macromolecules are secreted in a dry form and thus increase the concentration of the mucus gel layer if the availability of airway surface fluid is limited. In their gel-on-brush model, Button *et al*. recently showed that an increased concentration of mucins causes an increase in the osmotic pressure of the mucus gel layer and, above a certain threshold, this causes a compression of the subjacent periciliary layer, leading to a collapse of the cilia and mucostasis [6].

Since observational evidence indicates that there is a degree of airway surface dehydration in patients with COPD, this study aimed to investigate the *in vivo* effect of airway surface dehydration on several pathological hallmarks of COPD and on lung function. To achieve this goal, we exposed βENaC-Tg mice and wild-type (WT) littermates to air or CS for 4 or 8 weeks and determined mucin expression, goblet cell metaplasia, pulmonary inflammation, lymphoid follicles, pulmonary emphysema and airway wall remodelling, and measured lung function.

## Methods

Details of materials and methods used can be found in the online supplement (S1 File).

### Primary tracheal epithelial cultures

For each experiment, freshly excised tracheae were collected and pooled from 10 mice per group. Epithelial cells were isolated and cultured on membranes (T-Col, Costar, Cambridge, MA) under air-liquid interface conditions as described previously [22], and cultures were studied after reaching confluence (14 days).

### Measurement of airway surface liquid height

Primary tracheal epithelial cultures were washed with PBS, and 20 μl of PBS containing 2 mg/ml Rhodamine dextran (10 kDa; Molecular Probes) was added to the lumen to visualize the airway surface liquid layer. To avoid evaporation of the ASL, 80 μl of immiscible perfluorocarbon (Fluorinert-77, Sigma-Aldrich) was added to the airway surface following the addition of the labeling dye. Images of the Rhodamine-labeled airway surface liquid were acquired by confocal microscopy (Leica TCS SP8, Leica Microsystems, Mannheim, Germany). The height of the airway surface liquid was measured by averaging the heights obtained from *xz* scans of sixteen predetermined positions on the culture as previously described [22]. Airway surface liquid height was measured 5 min following the addition of the Rhodamine dextran and at designated time points over a period of 24 h in primary tracheal epithelial cultures from βENaC-Tg mice and WT littermates.

### Animals

Male βENaC-Tg mice, backcrossed onto the C57Bl/6 background [22], were mated with female C57Bl/6Cr1 wild-type (WT) mice (Charles River). All mice were bred in the animal facility of the Ghent University Hospital and housed in filtertop cages in standard conditions under a 12 h light-dark cycle and provided with a standard diet (Pavan, Brussels, Belgium) and chlorinated tap water *ad libitum*. Mice were euthanized with an overdose of pentobarbital (Sanofi, Libourne, France). All *in vivo* manipulations were approved by the local Ethics Committee for animal experimentation of the Faculty of Medicine and Health Sciences (Ghent University).

### Cigarette smoke exposure

Groups of 8 to 11 βENaC-Tg mice and WT littermates were exposed whole body to cigarette smoke as described before (a total of 120 mice was used) [23]. In short, mice were exposed 5 days a week to the mainstream cigarette smoke of 5 cigarettes (Reference cigarette 3R4F without filter, University of Kentucky, Lexington, KY, USA), 4 times a day with a 30 minute smoke free interval between exposures. A standard smoking apparatus was used with the smoking chamber adapted for a group of mice. A smoke/air ratio of 1/6 was obtained. Control mice were exposed to room air. CS exposure started at an age of 7–8 weeks and the exposure period was either 4 or 8 weeks.

### βENaC immunohistochemistry

Lung sections were evaluated for overexpression of the β-subunit of ENaC, using a rabbit polyclonal anti-βENaC antibody [25].

### Goblet cell analysis and mucin gene expression

Transversal sections were made from the paraffin-embedded left lung and were stained with Periodic acid-Schiff (PAS). Goblet cells were counted using Axiovision software (Zeiss) and were expressed as number of cells per millimetre basal membrane. Expression of Muc5ac and Muc5b was quantified by quantitative real-time polymerase chain reaction (qRT-PCR).

### Pulmonary inflammation

Bronchoalveolar lavage (BAL) fluid was collected via a tracheal cannula. Differential cell counts of the lavage fluid were obtained by cytocentrifuged preparations after May-Grünwald-Giemsa staining. Flow cytometric analysis was used for quantifying inflammatory cell types in BAL fluid and single cell suspensions of lung tissue [23–25]. qRT-PCR was used to evaluate the expression of several chemokines. The protein levels of Cxcl1 and Ccl20 in BAL fluid supernatant of mice were determined with an ELISA kit (R&D systems).

### Lymphoid follicles

In order to quantify lymphoid follicles, defined as dense accumulations of at least 50 lymphocytes, paraffin-embedded sections of the left lung were immunohistochemically stained with anti-CD3 (Dako) and anti-B220 (BD biosciences) [26]. The number of follicles was normalized for the total area of parenchyma that was scored.

### Emphysema

In order to evaluate pulmonary emphysema, two complementary methods were used, the mean linear intercept (Lm) and the destructive index (DI). The Lm is a measurement of alveolar space enlargement whereas the DI is a calculation of the percentage of destroyed alveolar walls. Both analyses were performed using the Image J software on haematoxylin and eosin (H&E) stained lung sections.

### Airway wall remodelling

To evaluate the deposition of fibronectin and collagen, paraffin-embedded sections of the left lung were used for immunohistological staining. Fibronectin was stained with mouse anti-fibronectin (Thermo-Scientific). Collagen was stained chemically with Sirius Red. The amount of collagen and fibronectin in the airway wall was quantified using the Axiovision software (Zeiss).

### Lung function measurements

Using the Flexivent System (SCIREQ, Montreal, Canada), baseline lung function was examined invasively in tracheostomised anaesthetized mice [27]. The jugular vein was used to administer pancuronium bromide (1 mg/kg) (Inresa, Freiburg, Germany), which induces a neuromuscular blockade. The mice were ventilated with an average breathing frequency of 150 breaths/minute. Once the mice were stable, resistance (R) and dynamic compliance (C_dyn_) were measured using a ‘snapshot perturbation’ manoeuvre. The forced oscillation perturbation (Quick Prime 3) was applied to assess the tissue damping (G). Pressure-volume (PV) loops were generated to measure the static compliance (C_stat_), total lung capacity (TLC) and hysteresis.

### Statistical analyses

Sigma Stat Software (SPSS 21.0, Chicago, IL, USA) was used to perform non-parametric tests (Kruskall-Wallis and Mann-Whitney-U). Reported values are expressed as mean ± SEM. P-values < 0.05 were considered to be significant.

## Results

### Overexpression of βENaC and reduced airway surface liquid height in βENaC-Tg mice

**Fig 1:**
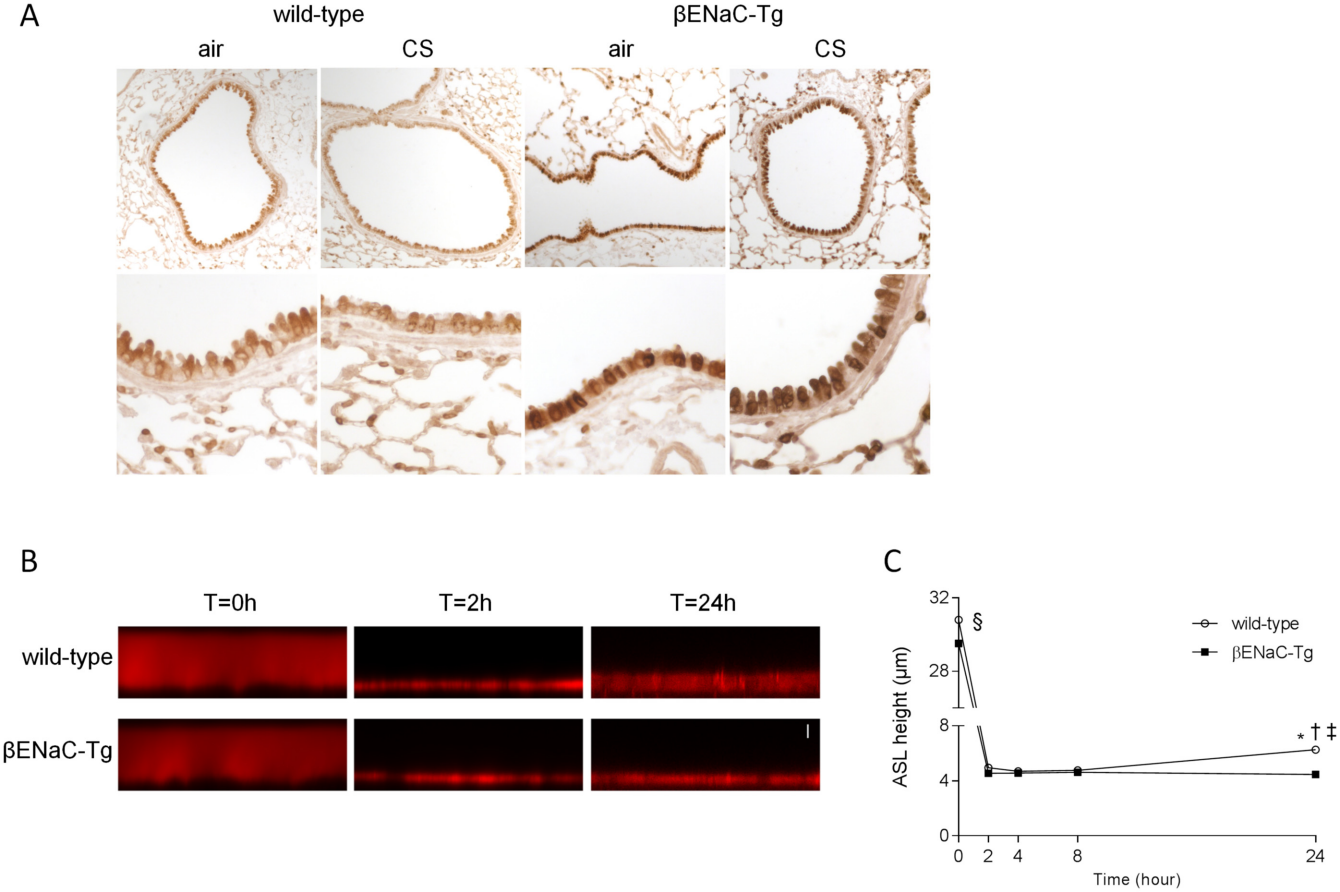
Overexpression of βENaC and reduced airway surface liquid height in βENaC-Tg mice. Immunolocalization of βENaC in airways from WT and βENaC-Tg mice. **(A)** Representative images of βENaC immunostaining of lung sections from WT and βENaC-Tg mice that were exposed to air or CS for 8 weeks. n = 5 per group. Dysregulation of steady state airway surface liquid (ASL) height on airway epithelia from βENaC-Tg mice under thin film conditions. Representative confocal images **(B)** and summary of measurements of airway surface liquid height **(C)** at t = 0, 2, 4, 8 and 24h after mucosal addition of 20 μl of PBS containing Rhodamine dextran to primary tracheal epithelial cultures from βENaC-Tg mice and WT littermates. Scale bar, 7 μm. n = 4 experiments per group. *p<0.001 compared to βENaC-Tg; ^§^p<0.001 for t = 0h compared to all other time points within the same genotype; ^†^p<0.05 for t = 24h wild-type compared to t = 2h wild-type; ^‡^p<0.005 for t = 24h wild-type compared to t = 4h and 8h wild-type.

To confirm the overexpression of the β-subunit of ENaC in βENaC-Tg mice, we performed an immunohistochemical staining for βENaC on lung tissue sections from WT and βENaC-Tg mice. βENaC-positive cells were readily detected in conducting airways and alveoli in lungs of WT mice (Fig 1A). The intensity of the immunoreactive signal was substantially stronger in airways from βENaC-Tg compared to WT mice, consistent with a marked increase in βENaC protein in epithelial cells (Fig 1A).

To determine the effects of βENaC overexpression on the regulation of airway surface liquid, primary tracheal epithelial cell cultures of WT and βENaC-Tg mice grown at an air-liquid-interface were challenged with a small volume of liquid added to the luminal compartment. Airway surface liquid height was monitored sequentially by confocal microscopy over a period of 24h. Within 2h after the initial volume challenge, the airway surface liquid was absorbed to a height of ~4.5 μm in both WT and βENaC-Tg mice (Fig 1B and 1C). However, at 24h, airway surface liquid height increased to ~6.3 μm in airway cultures from WT mice, whereas it remained significantly reduced in βENaC-Tg mice (Fig 1B and 1C). Consistent with previous studies [22], these results demonstrate that βENaC-Tg airway epithelia fail to regulate airway surface liquid to normal levels, and that steady state airway surface liquid is reduced in βENaC-Tg compared to WT mice.

### Cigarette smoke-induced mucin expression is aggravated in βENaC-Tg mice

**Fig 2:**
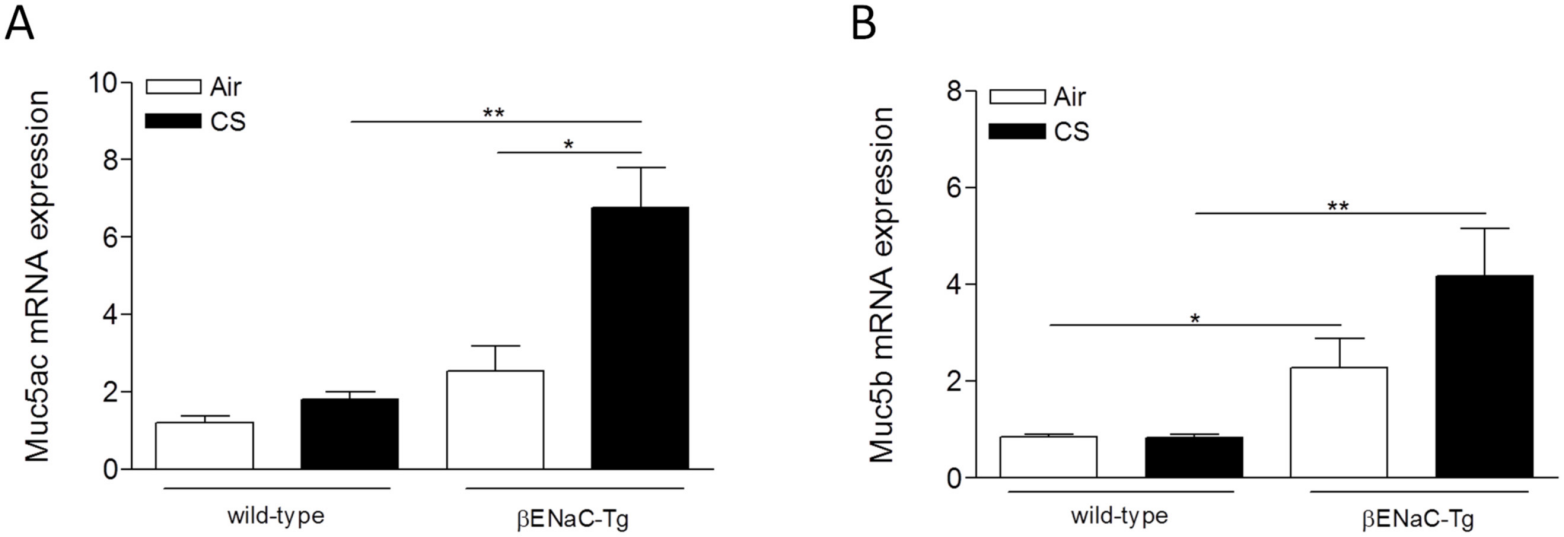
Cigarette smoke-induced mucin expression is increased in βENaC-Tg mice. mRNA expression of Muc5ac **(A)** and Muc5b **(B)** in total lung tissue upon 4 weeks of air or CS exposure. mRNA expression data were normalized for 3 reference genes (Hprt1, Gapdh, Tfrc). n = 6/group. *p<0.05, **p<0.01, ***p<0.001.

The expression of Muc5ac and Muc5b was quantified in total lung tissue by qRT-PCR. The airway surface dehydration in air-exposed βENaC-Tg mice resulted in a higher expression of both Muc5ac and Muc5b, compared to WT mice (Fig 2A and 2B). Four weeks of CS exposure significantly increased the expression of Muc5ac in βENaC-Tg mice, but not in WT mice (Fig 2A). In contrast, CS exposure did not induce a significant upregulation of Muc5b expression in lung, neither in βENaC-Tg mice nor in WT mice (Fig 2B). Similar results were obtained after 8 weeks of CS exposure (S1 Fig).

**Fig 3:**
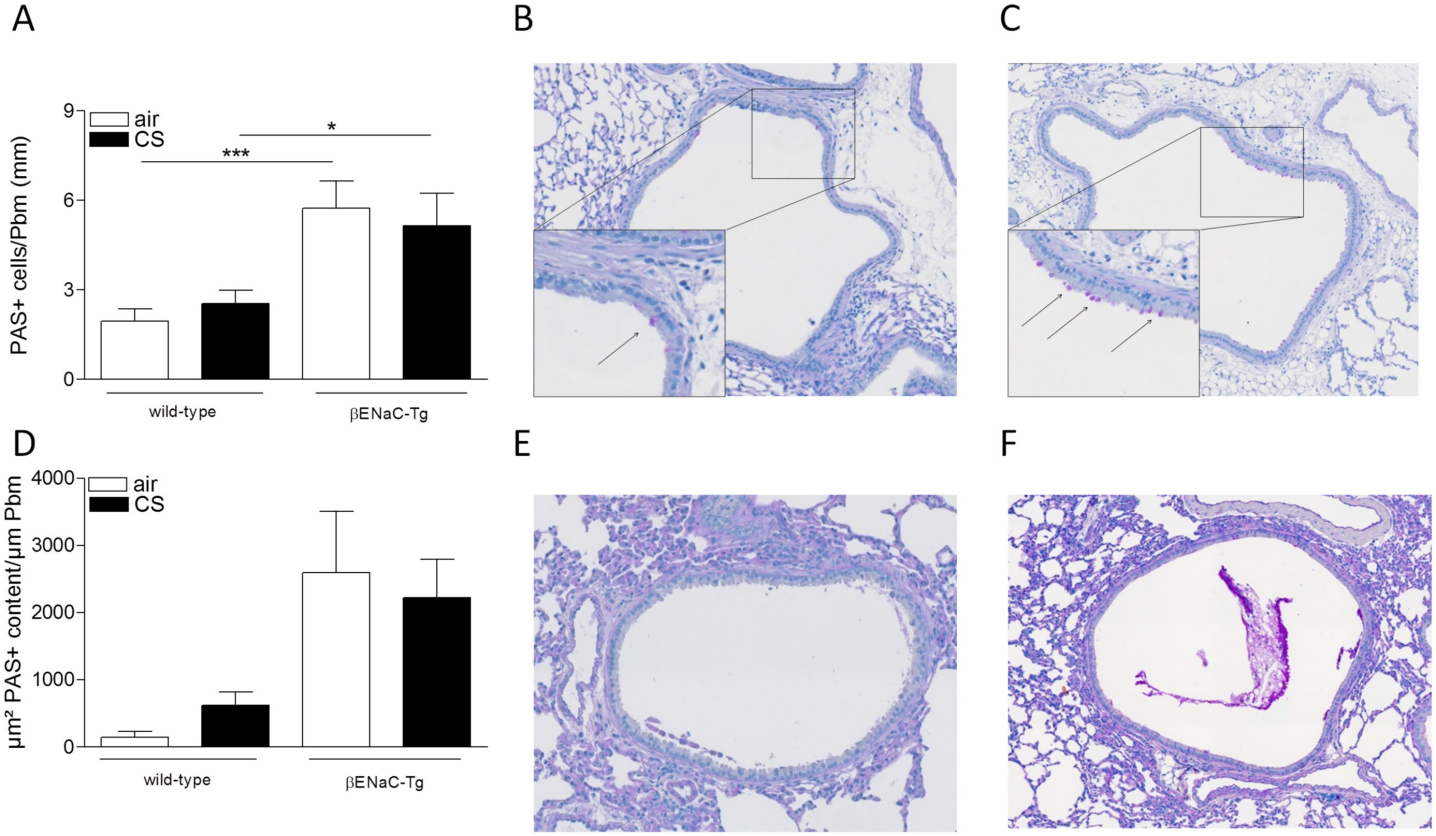
Goblet cell metaplasia and mucus secretion upon air or CS exposure. **(A)** Goblet cell count upon 4 weeks of air or CS exposure. n = 8/group. Representative images of goblet cells in airways of CS-exposed WT mice **(B)** and CS-exposed βENaC-Tg mice **(C)** upon 4 weeks of CS exposure. Arrows indicate goblet cells. **(D)** Quantification of PAS+ mucus content in lumen of airways of non-lavaged mice upon 4 weeks of air or CS exposure n = 3/group. Representative image of PAS+ mucus content in airways of CS-exposed WT mice **(E)** and CS-exposed βENaC-Tg mice **(F)**. *p<0.05, **p<0.01, ***p<0.001.

Goblet cell metaplasia was assessed by quantifying the number of periodic-acid-Schiff positive (PAS+) cells in the bronchial epithelium. Air-exposed βENaC-Tg mice had higher numbers of PAS+ cells compared to WT mice. However, 4 weeks of CS exposure did not induce an increase in PAS+ cells, neither in WT nor in βENaC-Tg mice (Fig 3A–3C). In a subgroup of animals (n = 3/group), PAS+ mucus content was measured in the airway lumen of non-lavaged mice following 4 weeks of air or CS exposure. Similar to goblet cell metaplasia, there was more mucus present in the airway lumen of βENaC-Tg mice, compared to WT controls independent of CS exposure (Fig 3D–3F). Quantification of goblet cell metaplasia after 8 weeks of CS exposure, resulted in a similar outcome (S1 Fig).

### Cigarette smoke-induced pulmonary inflammation is aggravated in βENaC-Tg mice

**Fig 4:**
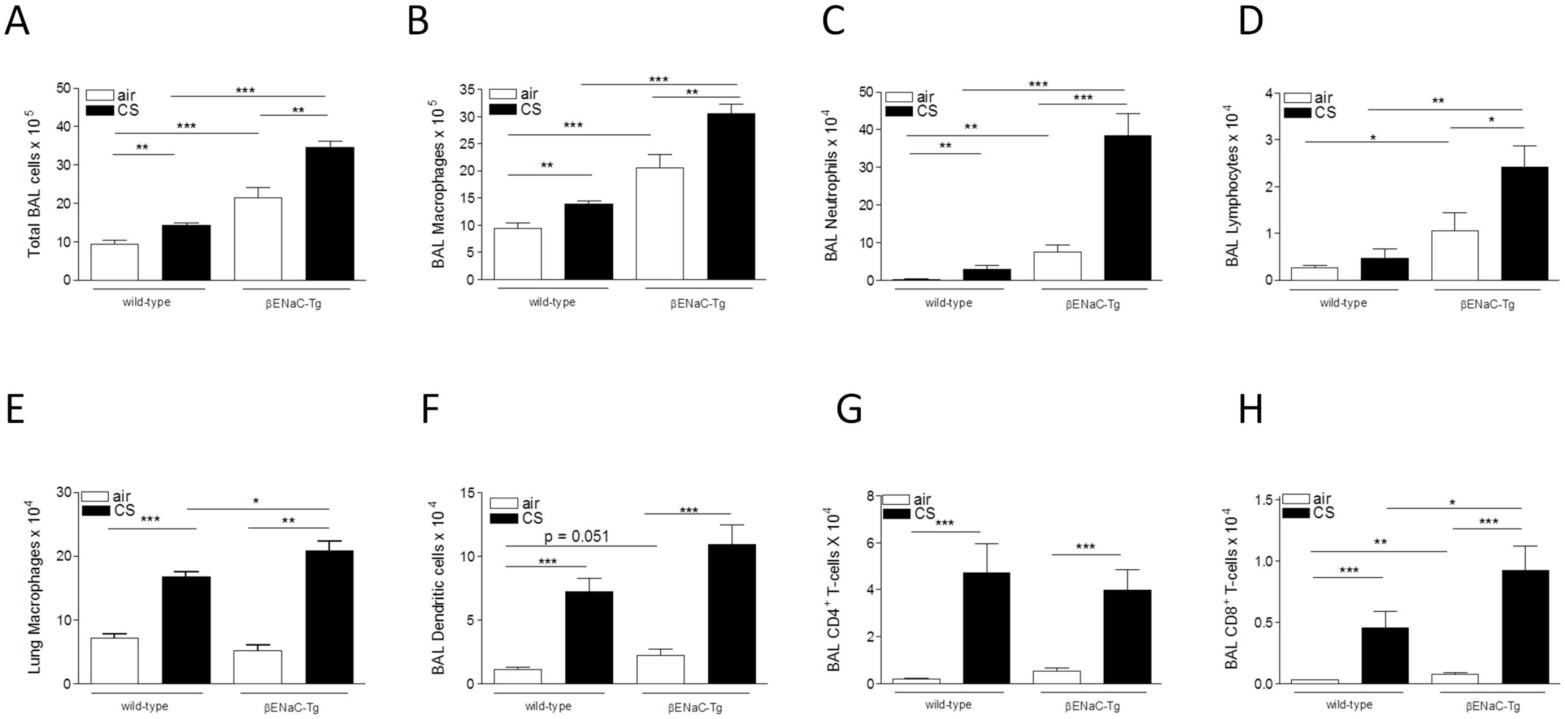
Cigarette smoke-induced pulmonary inflammation is increased in βENaC-Tg mice. **(A)** Total inflammatory cell count in BAL upon 4 weeks of air or CS exposure. Quantification of macrophages **(B)**, neutrophils **(C)** and lymphocytes **(D)** in BAL upon 4 weeks of air or CS exposure. n = 7-8/group. **(E)** Quantification of macrophages in total lung after 4 weeks of CS exposure. n = 7-8/group. Quantification of dendritic cells **(F)**, CD4+ T-lymphocytes **(G)** and CD8+ T-lymphocytes **(H)** in BAL upon 8 weeks of air or CS exposure. n = 8-11/group. *p<0.05, **p<0.01, ***p<0.001.

Following air exposure, the numbers of inflammatory cells in BAL fluid were significantly increased in βENaC-Tg compared to WT mice. Four weeks of CS exposure led to a significant increase in the number of total BAL cells, macrophages, neutrophils and lymphocytes, both in WT and βENaC-Tg mice (Fig 4A–4D). Importantly, this increase in innate and adaptive immune cells was significantly higher in βENaC-Tg mice, compared to WT mice (Fig 4A–4D). Additionally, 4 weeks of CS exposure significantly increased the number of macrophages in the lungs of βENaC-Tg and WT mice (Fig 4E), but had no effect on the number of neutrophils, dendritic cells and CD4+ and CD8+ T-lymphocytes (data not shown). Interestingly, the CS-induced increase in macrophages in lung tissue was aggravated in βENaC-Tg mice, compared to WT controls (Fig 4E).

Similar results were obtained after 8 weeks of CS exposure (S2 Fig). Following 8 weeks of CS exposure, additional cell types were quantified. While the CS-induced increase in dendritic cells and CD4+ T-lymphocytes in BAL did not differ in WT and β-ENaC (Fig 4F–4H), the increase in CD8+ T-lymphocytes was significantly aggravated in CS-exposed βENaC-Tg mice, compared to WT littermates (Fig 4H).

**Fig 5:**
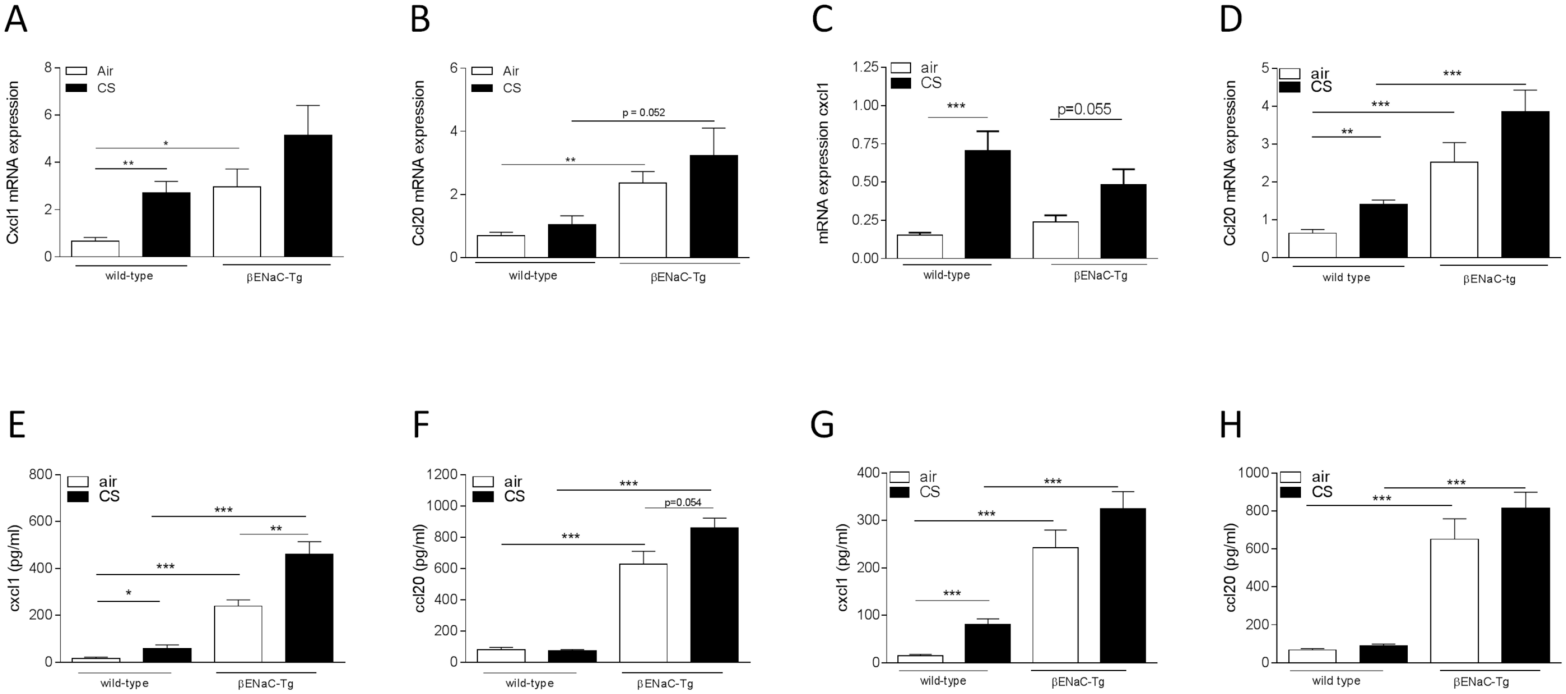
mRNA expression and protein levels of chemokines upon air or CS exposure. mRNA expression of Cxcl1 in total lung tissue upon 4 weeks **(A)** and 8 weeks **(C)** air or CS exposure. mRNA expression of Ccl20 in total lung tissue upon 4 weeks **(B)** and 8 weeks **(D)** air or CS exposure. mRNA expression data were normalized for 3 reference genes (Hprt1, Gapdh, Tfrc). n = 6-8/group. Protein levels of Cxcl1 in BAL fluid upon 4 weeks **(E)** and 8 weeks **(G)** air or CS exposure. Protein levels of Ccl20 in BAL fluid upon 4 weeks **(F)** and 8 weeks **(H)** air or CS exposure. Protein levels were measured by ELISA. n = 8-11/group. *p<0.05, **p<0.01, ***p<0.001.

Quantification of inflammatory chemokines revealed higher mRNA expression of Cxcl1 and Ccl20 in lung tissue of air-exposed βENaC-Tg mice, compared to WT littermates (Fig 5A–5D). The CS-induced increase in pulmonary Ccl20 mRNA expression was aggravated in βENaC-Tg mice, especially upon 8 weeks of CS exposure (Fig 5A–5D). Protein levels of Cxcl1 and Ccl20 in BAL fluid were significantly higher in βENaC-Tg mice, compared to WT littermates (Fig 5E–5H). Moreover, the CS-induced increase in Cxcl1 protein levels in BAL was significantly aggravated in βENaC-Tg mice (Fig 5A–5D).

### Cigarette smoke-induced formation of lymphoid follicles is accelerated in βENaC-Tg mice

**Fig 6:**
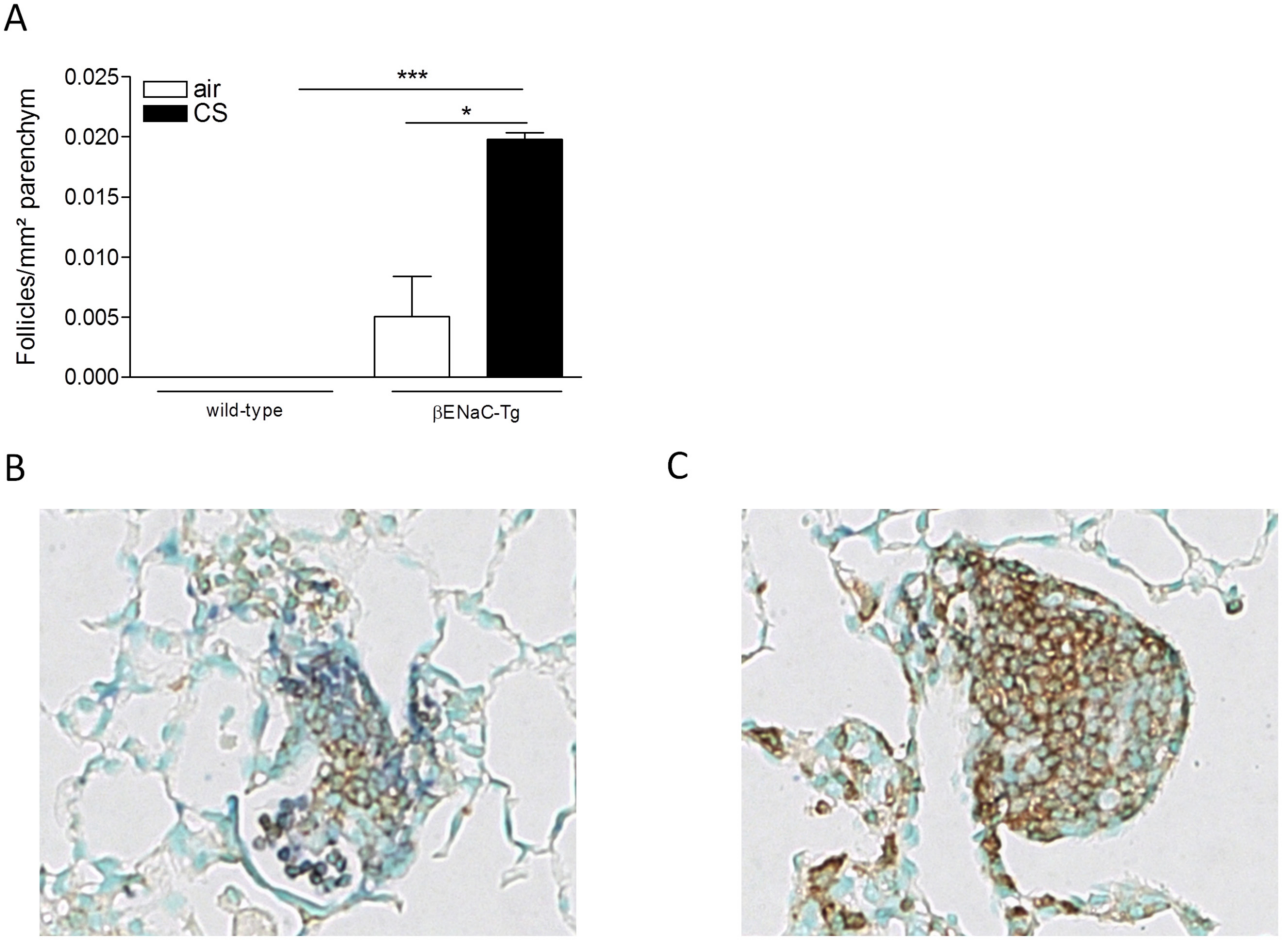
Cigarette smoke-induced lymphoid follicle formation in βENaC-tg, but not in WT mice after 8 weeks of CS exposure. **(A)** Quantification of lymphoid follicles normalized for the area of parenchyma (mm^2^) upon 8 weeks of air or CS exposure. n = 8-11/group. **(B–C)** Representative images of lymphoid follicles found in CS-exposed βENaC-Tg mice. *p<0.05, **p<0.01, ***p<0.001.

Eight weeks of CS exposure did not induce lymphoid follicles in WT mice. In contrast, after 8 weeks of CS exposure, the formation of lymphoid follicles was already detected in βENaC-Tg mice (Fig 6). Whereas lymphoid follicle formation upon chronic CS exposure (i.e. 6 months) was shown to be associated with elevated expression of Cxcl13 in WT mice, transcript levels of this chemokine were not increased following 8 weeks of CS-exposure in βENaC-Tg mice (data not shown).

### Cigarette smoke-induced alveolar destruction is aggravated in βENaC-Tg mice

**Fig 7:**
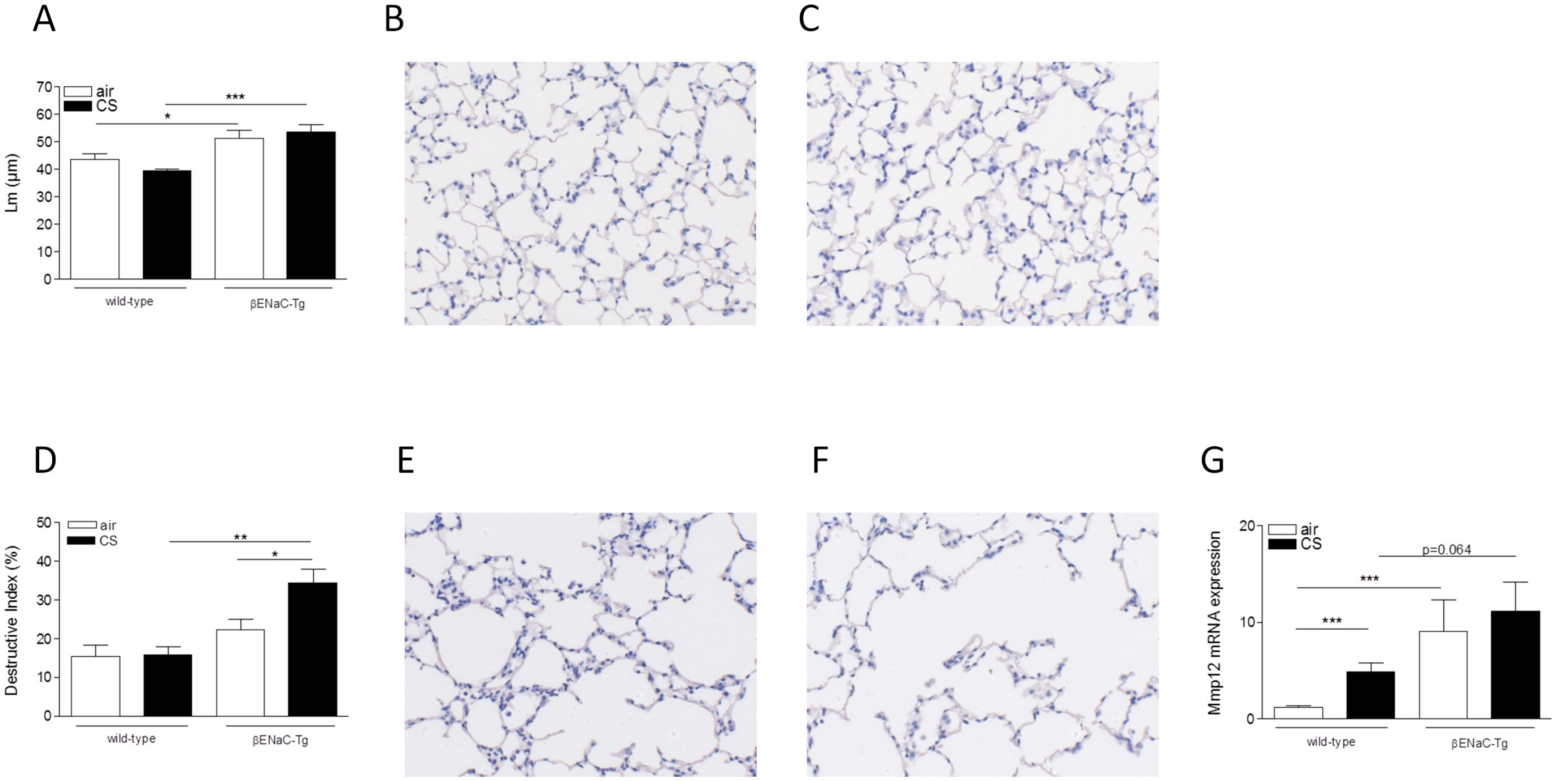
Cigarette smoke-induced alveolar destruction is increased in βENaC-Tg mice. **(A)** Mean linear intercept (Lm) upon 4 weeks of air or CS exposure. n = 7-8/group. Representative image of WT mice: air-exposed **(B)** and CS-exposed **(C)**. Destructive index (DI) upon 4 weeks of air or CS exposure **(D)**. n = 7-8/group. Representative image of βENaC-Tg mice: air-exposed **(E)** and CS-exposed **(F)**. mRNA expression of Mmp12 in total lung upon 4 weeks of air- or CS exposure **(G)**. Normalized for 3 reference genes (Hprt1, Gapdh, and Tfrc). n = 6/group. *p<0.05, **p<0.01, ***p<0.001.

Emphysema was measured by two complementary methods. The mean linear intercept (Lm), a measure for alveolar airspace enlargement, was significantly enlarged in the βENaC-Tg mice compared to WT littermates (Fig 7A). However, 4 weeks of CS exposure did not further increase the Lm, neither in the βENaC-Tg mice nor in the WT controls (Fig 7A). Emphysema was also quantified by determining the destructive index (DI). This measure quantifies the percentage of destruction of the alveolar walls. The DI tended to be higher in air-exposed βENaC-Tg compared to WT mice (Fig 7D). Importantly, 4 weeks of CS exposure did not result in an increased DI in WT mice, but did induce a significantly increased level of destruction in βENaC-Tg mice. (Fig 7D). Similar results were obtained after 8 weeks of CS exposure (S3 Fig). Interestingly, Mmp12 mRNA expression was higher in lungs of βENaC-Tg mice compared to WT littermates (Fig 7G).

### Cigarette smoke does not induce airway wall remodelling in βENaC-Tg mice

We investigated airway wall remodelling in mice by measuring the amount of fibronectin and collagen deposited in the airway walls. Fibronectin and collagen deposition in the airway walls did not differ between WT and βENaC-Tg mice and was not affected by 4 or 8 weeks of CS exposure (S4 Fig).

### Cigarette smoke-induced changes in lung function in WT and βENaC-Tg mice

**Fig 8:**
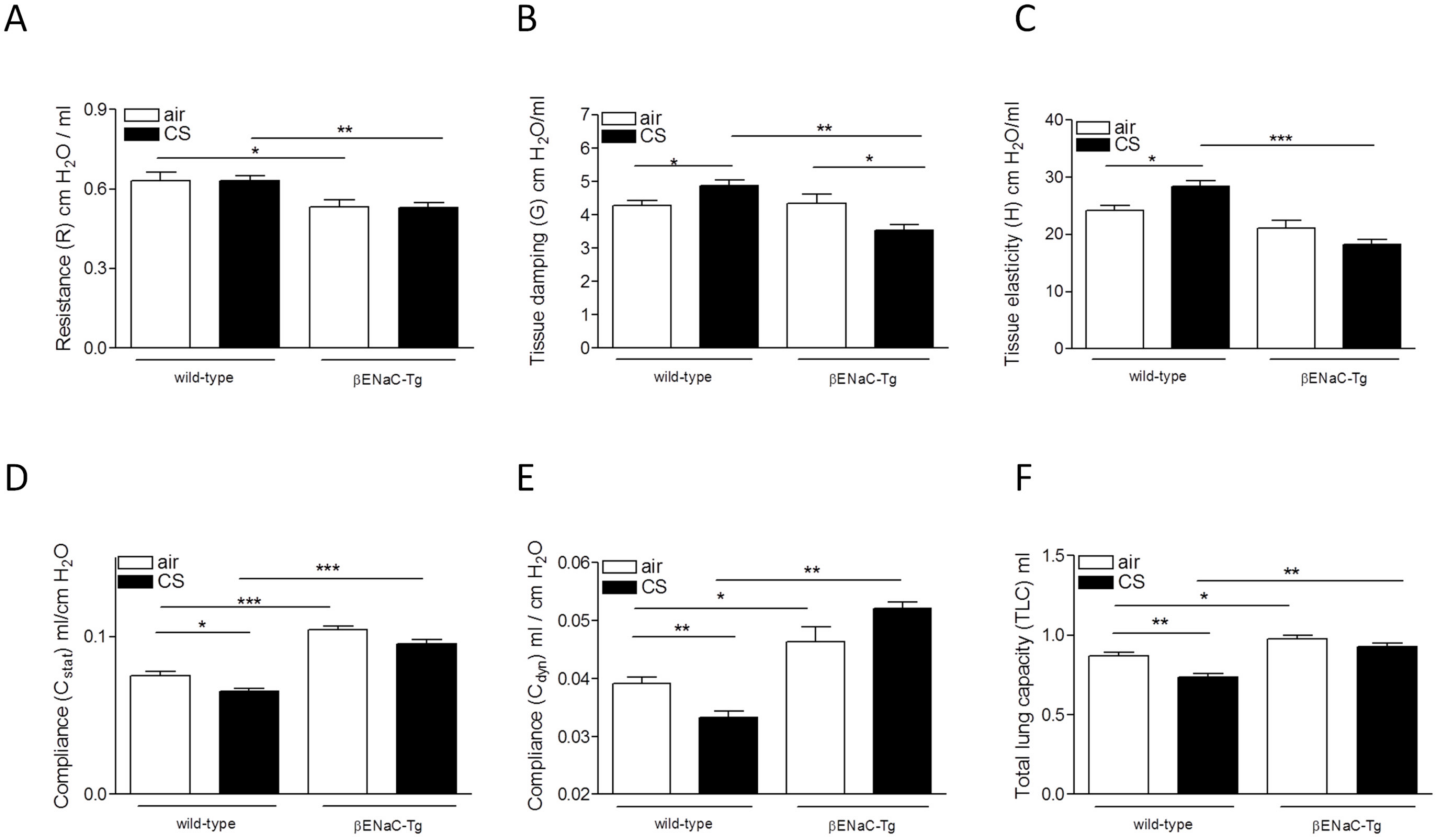
Effect of CS exposure on lung function in WT and βENaC-Tg mice. Lung function was determined in WT and βENaC-Tg mice after exposure to air or CS for 4 weeks. **(A)** Resistance (R) of the entire compartment (airways, tissue and chest wall). **(B)** Tissue damping (G), related to tissue resistance. **(C)** Tissue elasticity (H). **(D)** Static compliance (C_stat_). **(E)** Dynamic compliance (C_dyn_). **(F)** Total lung capacity (TLC). *p<0.05, **p<0.01, ***p<0.001.

We assessed the pulmonary function of WT and βENaC-Tg mice after 4 weeks of exposure to air or CS. Air-exposed βENaC-Tg mice exhibited a lower total pulmonary resistance, compared to air-exposed WT mice (Fig 8A). CS exposure did not influence the total pulmonary resistance, neither in WT nor in βENaC-Tg mice (Fig 8A). However, CS exposure significantly decreased the tissue damping in βENaC-Tg mice, whereas CS exposure of WT mice resulted in an increased tissue damping (Fig 8B). This parameter is used to assess the tissue resistance. In addition, the tissue elasticity was significantly lower in CS-exposed βENaC-Tg mice, compared to CS-exposed WT mice (Fig 8C).

**Fig 9:**
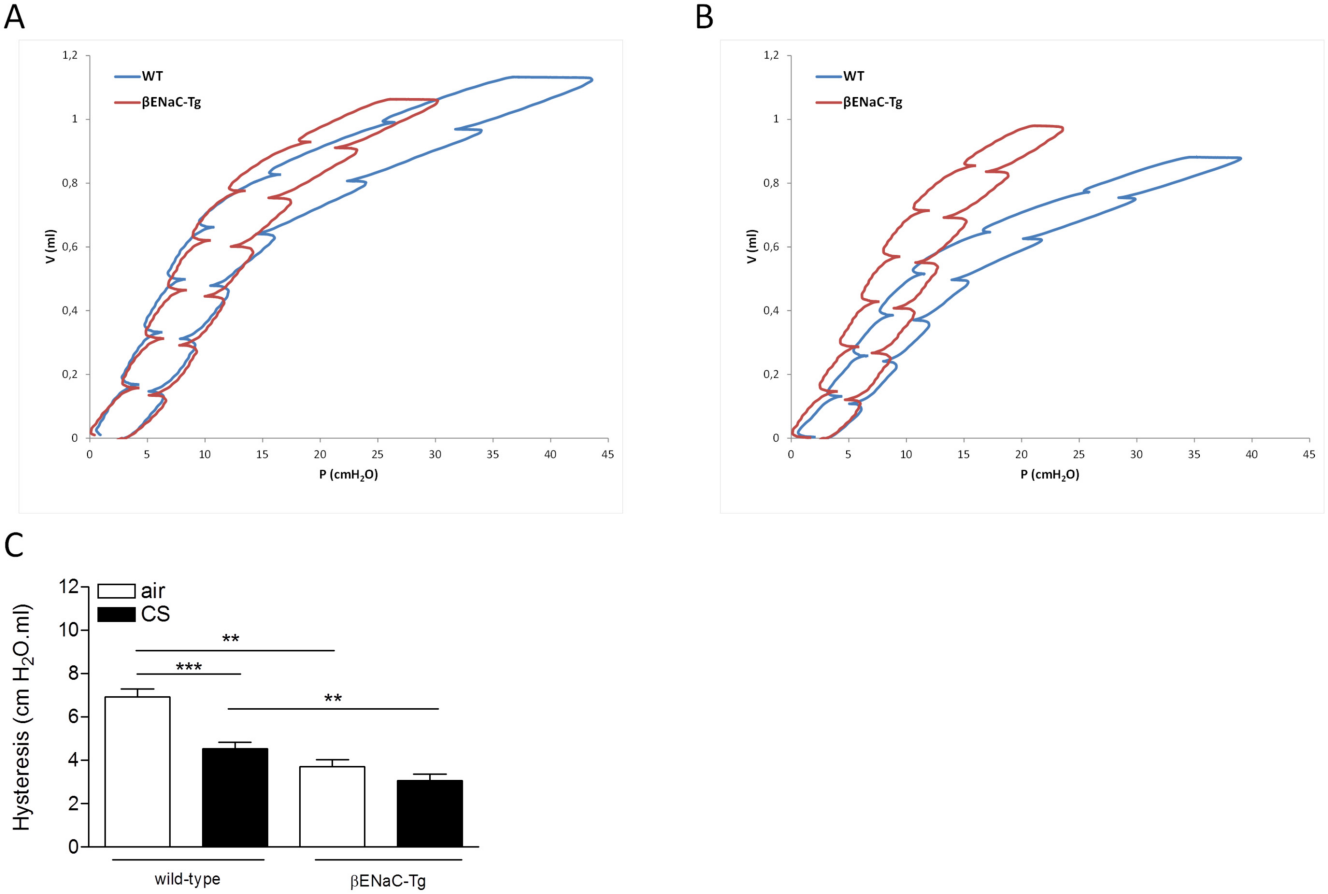
Pressure-Volume loops of air- and CS-exposed mice. **(A)** Representative PV loops of air-exposed WT (black) and βENaC-Tg (red) mice. **(B)** Representative PV loops of CS-exposed WT (black) and βENaC-Tg (red) mice. **(C)** Area of PV loop or hysteresis. *p<0.05, **p<0.01, ***p<0.001.

A significantly increased static and dynamic compliance was registered in βENaC-Tg mice compared to WT mice, consistent with the increase in mean linear intercept measured in βENaC-Tg mice (Fig 8A). Whereas CS exposure induced a significant decrease of both compliances in WT mice, CS exposure had no significant effect on the elevated static and dynamic compliance in the βENaC-Tg mice (Fig 8D and 8E). The total lung capacity (TLC) was significantly higher in βENaC-Tg compared to WT mice. CS exposure induced a decrease in TLC in WT mice, but did not influence this parameter in βENaC-Tg mice (Fig 8F). Analysis of the PV-loops demonstrated that the hysteresis, i.e. area between inflating and deflating part of the PV-loop, was significantly decreased in air-exposed βENaC-Tg mice compared to WT mice (Fig 9A–9C). Exposing mice to CS, decreased the hysteresis both in WT and in βENaC-Tg mice. Of note, hysteresis was significantly lower in CS-exposed βENaC-Tg mice compared to CS-exposed WT mice (Fig 9C).

## Discussion

There is increasing evidence of airway surface dehydration in smokers and patients with COPD. This study demonstrates that airway surface dehydration in βENaC-Tg mice aggravates CS-induced airway inflammation, mucin expression and destruction of alveolar walls and accelerates the formation of pulmonary lymphoid follicles. In contrast, CS exposure did not induce airway wall remodelling and had no effect on goblet cell metaplasia in βENaC-Tg mice.

It has been demonstrated that CS suppresses the function of the CFTR channel, with airway surface dehydration and decreased mucociliary clearance as consequences [8–10, 15, 28]. Moreover, Dransfield *et al*. showed that a diminished CFTR function correlates with the presence of chronic bronchitis and the degree of dyspnoea [15]. Since Cftr-deficient mice do not exhibit imbalanced airway ion transport in the lower airways and do not display cystic fibrosis-like disease [7, 28–31], we used βENaC-Tg mice, backcrossed onto a C57Bl/6 background [22], to test the hypothesis whether the presence of airway surface dehydration has an impact on CS-induced pathology and pathophysiology *in vivo*. βENaC-Tg mice overexpress the β-subunit of ENaC through an airway-specific club cell secretory protein (CCSP) promotor [7]. The constitutive hyperabsorption of Na^+^ leads to airway surface dehydration and decreased mucociliary clearance [7, 18].

Besides an imbalance of epithelial ion transport, mucus hypersecretion may contribute to airway surface dehydration and mucociliary dysfunction. As Button *et al*. elegantly showed in their gel-on-brush model, a hyperconcentrated mucus gel layer with > 8% solids can create sufficient osmotic pressure to cause a collapse of the cilia and mucostasis [6]. Clunes *et al*. found that mucus of chronic cigarette smokers contained approximately 10% solids, which makes it likely that osmotic pressure of the hyperconcentrated mucus layer contributes to mucociliary dysfunction in smokers [4, 32]. The predominant secreted mucins in airway mucus are MUC5AC and MUC5B and both are upregulated in COPD [19–21]. Consistent with previous studies, we observed higher transcript levels of Muc5ac and Muc5b and a significant higher number of goblet cells in βENaC-Tg mice compared to WT mice [18]. Although the Muc5b mRNA expression tended to be increased after 4 weeks of CS exposure, this increase did not reach significance. In contrast, CS induced a significant increase of Muc5ac expression in the βENaC-Tg mice, both after 4 and 8 weeks of CS exposure. Since mice do not readily develop goblet cell metaplasia upon CS exposure [33, 34], we did not observe an increase in goblet cell metaplasia after CS exposure, neither in WT nor in βENaC-Tg mice. However, substantial mucin hypersecretion has been detected without increase in goblet cell numbers. It has been suggested that Clara cells produce and secrete Muc5ac without being subjected to metaplastic changes, including the formation of PAS positive storage granules [35, 36]. Taken together, our results are consistent with the observation that Muc5ac is highly inducible by noxious stimuli [20] and suggest that hypersecretion of Muc5ac in response to CS exposure may contribute to mucus hyperconcentration and airway surface dehydration.

Airway surface dehydration produces chronic airway inflammation in BAL in βENaC-Tg mice, with higher numbers of macrophages, neutrophils and lymphocytes compared to WT littermates [7, 18, 22]. Our study confirms and extends these findings by showing that BAL of βENaC-Tg mice also exhibits more dendritic cells and CD8+ T-lymphocytes. By exposing both WT littermates and βENaC-Tg mice to CS, we demonstrated that airway surface dehydration was associated with an aggravated CS-induced inflammatory response in βENaC-Tg mice. In BAL, CS exposure induced a significantly higher increase in macrophages, neutrophils and CD8^+^ T-lymphocytes, key players in COPD pathophysiology, in βENaC-Tg mice than in WT mice [13, 37–39]. Interestingly, this exaggerated response already occurred after a subacute CS exposure, i.e. 4 weeks.

In lung tissue, the CS-induced inflammatory response was limited to an increase in macrophages. Along with this increase in macrophage numbers, we observed an increase in matrix metalloproteinase 12 (Mmp12), a macrophage-derived protease important in emphysema development in WT and βENaC-Tg mice [40, 41].

Severe COPD is associated with increased numbers of airways containing lymphoid follicles [12]. Moreover, the presence of lymphoid follicles has also been demonstrated in the lung parenchyma of patients with COPD [42]. In WT mice, lymphoid follicles are usually detected after 6 months of CS exposure [43]. In this study, lymphoid follicles were already detected in βENaC-Tg mice after 8 weeks of CS exposure, suggesting that airway surface dehydration accelerates the CS-induced formation of lymphoid follicles. Since we did neither observe a dominance of B cells in these lymphoid follicles, nor an upregulation in total lung tissue of Cxcl13 transcript levels, a chemokine involved in lymphoid follicle formation upon chronic CS exposure [43], we speculate that we observed early stages of follicle formation. Importantly, we did not find evidence of pulmonary infection.

In COPD patients, emphysema develops after many years of cigarette smoking and it consistently requires several months of CS exposure, i.e. 6 months, to evoke emphysema in WT mice. In contrast, βENaC-Tg mice develop severe emphysema early in life [18, 40, 44]. Similar elements that lead to the onset of emphysema, can be found in both βENaC-Tg mice and patients with COPD. First, in COPD, a proteinase/antiproteinase imbalance plays a key role in the development of emphysema [45–47]. Recently, it has been shown that Mmp12 and neutrophil elastase also mediate emphysema in βENaC-Tg mice [40, 48]. Second, McDonough *et al*. showed that small airway obstruction precedes emphysematous destruction in COPD patients [49]. In βENaC-Tg mice, airway surface dehydration leads to mucus obstruction in the first days of life and preceding the onset of emphysema [7, 18, 22], constituting another similarity to COPD patients. In this study, we confirmed severe emphysema in air-exposed βENaC-Tg mice, as quantified by the mean linear intercept (Lm) [18]. However, we did not find a further increase in Lm in the βENaC-Tg mice following CS exposure. Given the severity of the constitutive emphysema in adult βENaC-Tg mice, it can be questioned whether it is at all possible to establish further enlargement of the Lm upon CS exposure. In contrast, quantifying the destructive index (DI), a measure for alveolar destruction, clearly showed that 4 weeks of CS exposure already induced significant destruction of alveolar tissue in βENaC-Tg mice, while there was not yet an effect in WT mice. We also performed lung function measurements and observed a decreased resistance and increased compliance and TLC, indicating that the lung function is dominated by the severe spontaneous emphysema in βENaC-Tg mice [18]. Interestingly, following CS exposure, the increased compliance measured in βENaC-Tg mice was accompanied by a decreased tissue damping and tissue elasticity. Together with the morphometric analysis of the DI, these data demonstrate that airway surface dehydration aggravated CS-induced emphysema, even after short term exposure, suggesting that this mechanism may contribute to emphysema formation in smokers with COPD.

This study suggests that therapeutic targeting of airway surface dehydration may be beneficial in patients with COPD, although extrapolation of our experimental findings in mice after (sub)acute cigarette smoke-exposure to the complex chronic disease COPD in humans should be performed with caution. It has been shown *in vitro* that the CFTR potentiator ivacaftor can partially rescue the CS-induced CFTR deficiency, thereby restoring the airway surface dehydration and mucociliary clearance in non-CF epithelial cells [10]. Moreover, the phosphodiesterase inhibitor roflumilast has beneficial effects on the CS-induced dehydration of the airway surface of epithelial cell lines through elevation of intracellular cAMP levels and activation of CFTR [50]. These proof-of-concept studies may facilitate future clinical trials that will be required to determine therapeutic effects improving CFTR function and airway surface hydration on mucus obstruction, airway inflammation and emphysema in patients with COPD.

Of note, our results also suggest that CS exposure of βENaC-Tg mice can be used as a time-saving model for COPD-like pathology. Within a short time frame of 4 to 8 weeks, these mice already develop a strong pulmonary inflammation, with the formation of lymphoid follicles, and destruction of alveolar tissue, whereas these pathologies in WT mice can only be observed following 6 months of CS exposure. In addition, βENaC-Tg mice also possess more characteristics of chronic bronchitis, including goblet cell metaplasia and intraluminal mucus content, than WT mice.

In summary, acquired CFTR malfunction and mucin hypersecretion, both leading to mucus hyperconcentration, have been demonstrated in smokers with and without COPD, implicating that airway surface dehydration is present in these patients [8, 9]. In our study, we have shown that the presence of airway surface dehydration in βENaC-Tg mice significantly aggravates the CS-induced pathological hallmarks of COPD, including mucin expression, pulmonary inflammation, formation of lymphoid follicles and destruction of alveolar walls. We speculate that the impaired mucociliary clearance caused by airway surface dehydration results in a retention and concentration of CS in the airways, thus leading to exaggerated host responses, such as mucus hypersecretion and enhanced inflammation. Proteases, originating from macrophages and neutrophils, can then lead to destruction of alveolar tissue and emphysema. The results of our study identify airway surface dehydration as a novel risk factor for CS-induced pathology.

## Supporting Information

S1 Fig Goblet cell metaplasia and mucin expression upon 8 weeks of air or CS-exposure. **(A)** Goblet cell count. n = 8-11/group. **(B)** mRNA expression of Muc5ac. **(C)** mRNA expression of Muc5b. Expression data normalized for 3 household genes (Hprt1, Gapdh, and Tfrc). n = 8/group. *p<0.05, **p<0.01, ***p<0.001.
(TIF)

S2 Fig CS-induced inflammation in BAL upon 8 weeks of CS exposure. **(A)** Total inflammatory cell count in BAL. **(B)** Number of macrophages in BAL. **(C)** Number of neutrophils in BAL. **(D)** Number of lymphocytes in BAL. n = 8-11/group. *p<0.05, **p<0.01, ***p<0.001.
(TIF)

S3 Fig Cigarette smoke-induced alveolar destruction is increased in βENaC-Tg mice after 8 weeks of air or CS exposure. **(A)** Mean linear intercept (Lm) after 8 weeks of air or CS exposure. **(B)** Destructive index (DI) after 8 weeks of air of CS exposure. n = 8-11/group.
(TIF)

S4 Fig 8 weeks of cigarette smoke exposure does not induce airway wall remodelling in WT and βENaC-Tg mice. **(A)** Deposition of fibronectin in the airway wall. Normalized for perimeter basement membrane. **(B)** Deposition of collagen in the airway wall. Normalized for perimeter basement membrane. n = 8-11/group.
(TIF)

S1 File Extended material and methods. (DOCX)
