# Supplementary figures and images for: Airway Surface Dehydration Aggravates Cigarette Smoke-Induced Hallmarks of COPD in Mice
Source: PLoS One. 2015 Jun 12;10(6):e0129897. doi: 10.1371/journal.pone.0129897 (PMC4466573; doi:10.1371/journal.pone.0129897)

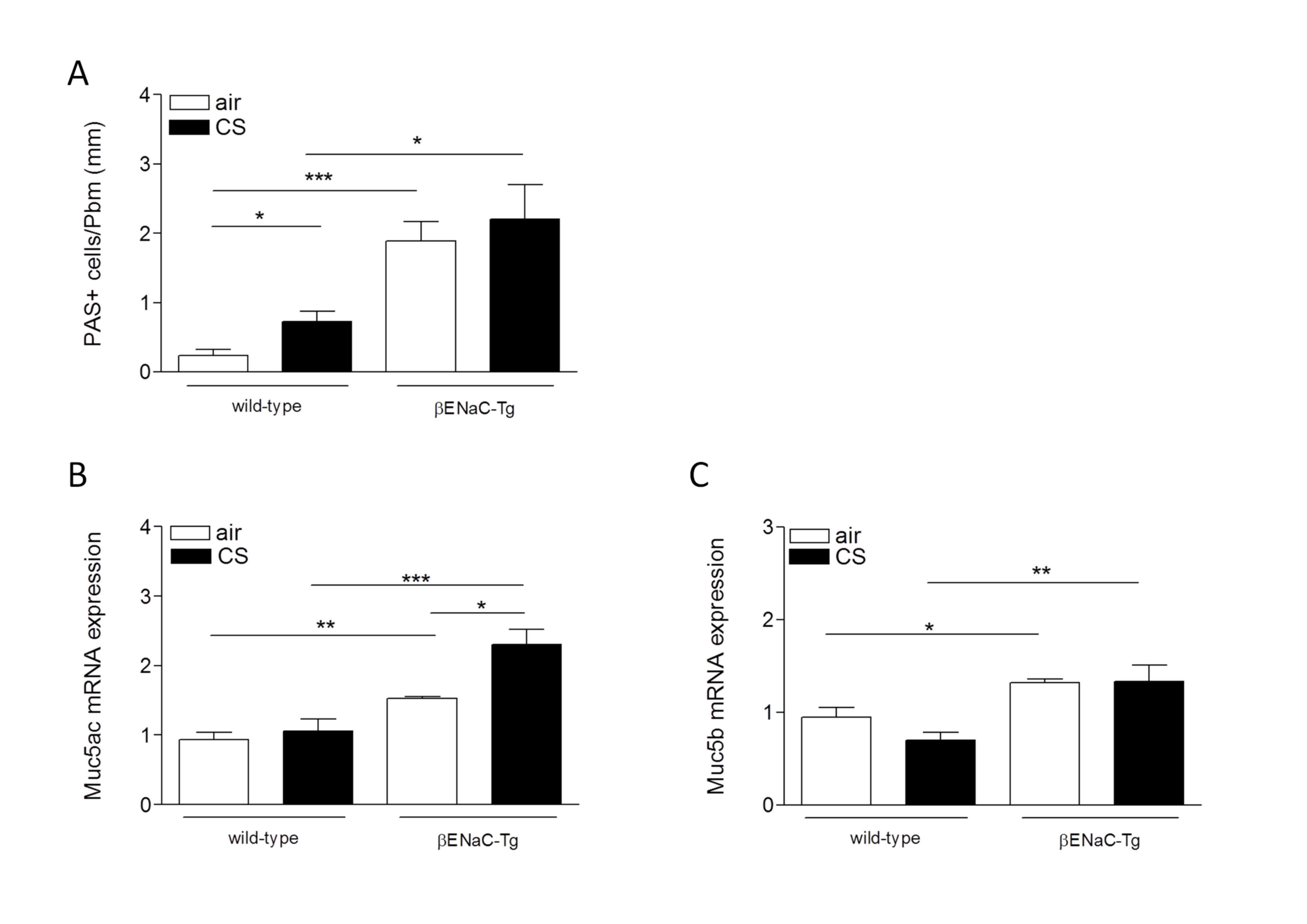

Supplement: S1 Fig — (A) Goblet cell count. n = 8-11/group. (B) mRNA expression of Muc5ac. (C) mRNA expression of Muc5b. Expression data normalized for 3 household genes (Hprt1, Gapdh, and Tfrc). n = 8/group. *p<0.05, **p<0.01, ***p<0.001. (TIF) [file pone.0129897.s001.tif]

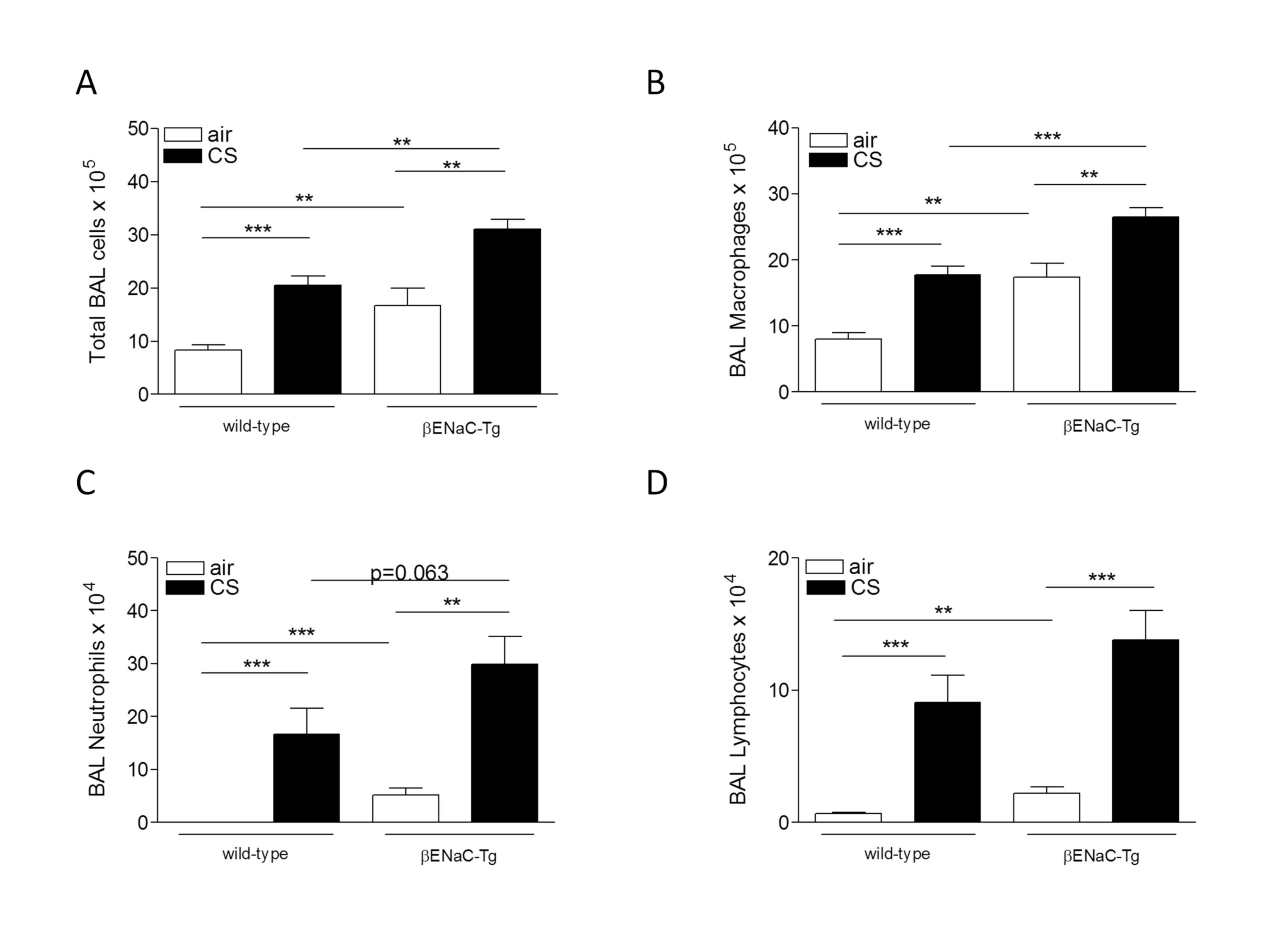

Supplement: S2 Fig — (A) Total inflammatory cell count in BAL. (B) Number of macrophages in BAL. (C) Number of neutrophils in BAL. (D) Number of lymphocytes in BAL. n = 8-11/group. *p<0.05, **p<0.01, ***p<0.001. (TIF) [file pone.0129897.s002.tif]

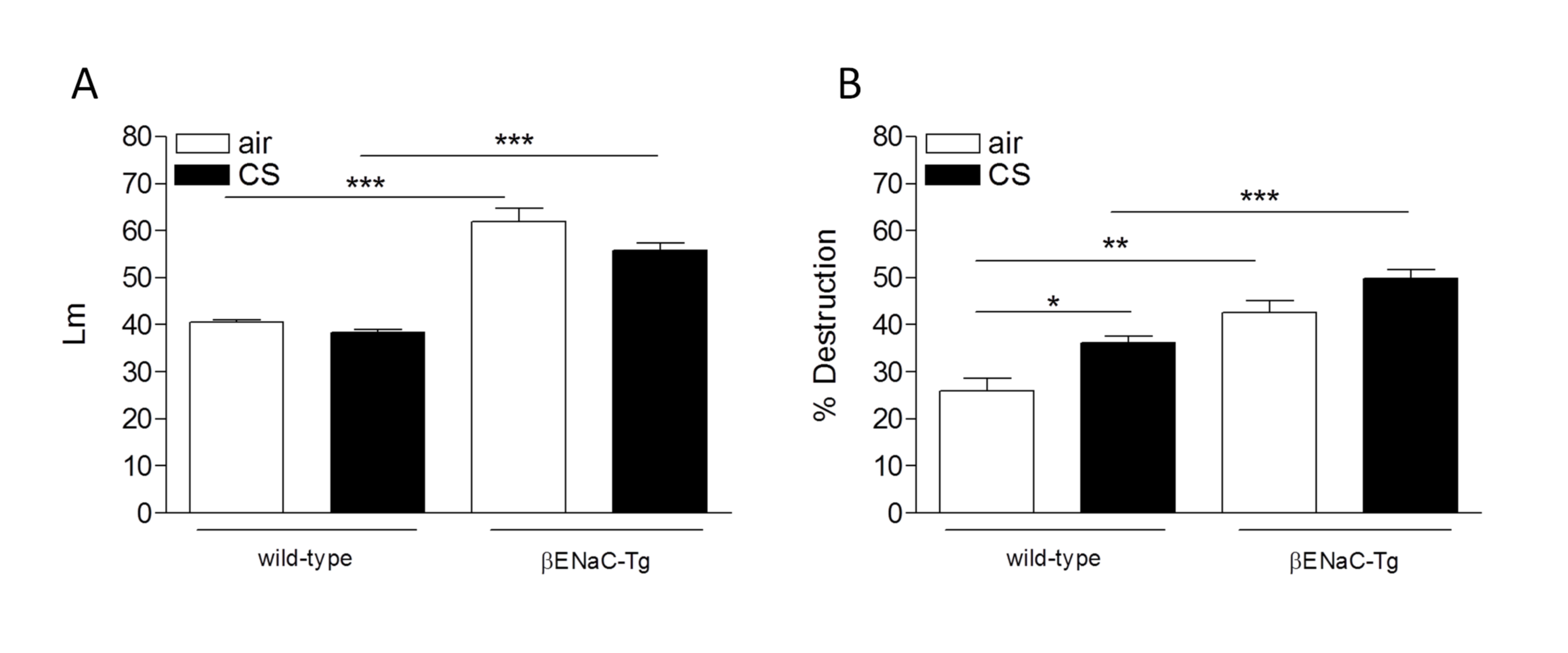

Supplement: S3 Fig — (A) Mean linear intercept (Lm) after 8 weeks of air or CS exposure. (B) Destructive index (DI) after 8 weeks of air of CS exposure. n = 8-11/group. (TIF) [file pone.0129897.s003.tif]

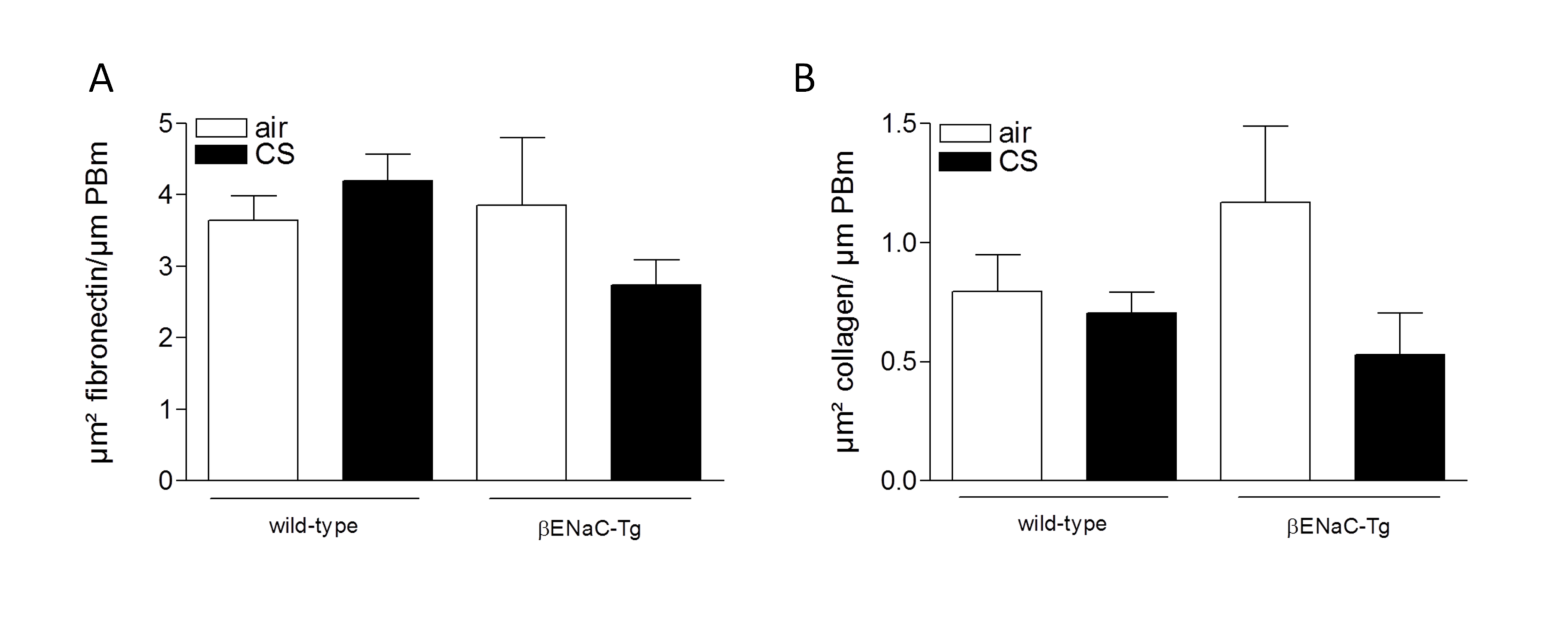

Supplement: S4 Fig — (A) Deposition of fibronectin in the airway wall. Normalized for perimeter basement membrane. (B) Deposition of collagen in the airway wall. Normalized for perimeter basement membrane. n = 8-11/group. (TIF) [file pone.0129897.s004.tif]
