# Supplementary material for: Airway Surface Dehydration Aggravates Cigarette Smoke-Induced Hallmarks of COPD in Mice
Source: PLoS One. 2015 Jun 12;10(6):e0129897. doi: 10.1371/journal.pone.0129897 (PMC4466573; doi:10.1371/journal.pone.0129897)
Supplement: S1 File — (DOCX) [file pone.0129897.s005.docx]

**Online supplement**

**Materials and methods**

**Primary Tracheal Epithelial Cultures**

For each experiment, freshly excised tracheae were collected and pooled from 10 mice per group. Epithelial cells were isolated and cultured on membranes (T-Col, Costar, Cambridge, MA) under air-liquid interface conditions as described previously (1), and cultures were studied after reaching confluence (14 days).

**Measurement of airway surface liquid height**

Primary tracheal epithelial cultures were washed with PBS, and 20 µl of PBS containing 2 mg/ml Rhodamine dextran (10 kDa; Molecular Probes) was added to the lumen to visualize the airway surface liquid layer. To avoid evaporation of the airway surface liquid, 80 µl of immiscible perfluorocarbon (Fluorinert-77, Sigma-Aldrich) were added to the airway surface following the addition of the labeling dye (2). Adding this volume of PBS resulted in an initial ASL height of 25–35 µm. Images of the Rhodamine-labeled airway surface liquid were acquired by confocal microscopy (Leica TCS SP8, Leica Microsystems, Mannheim, Germany) using the appropriate settings for Rhodamine (excitation with 561 nm laser/ emission detection at 600-650 nm). The height of the airway surface liquid was measured by averaging the heights obtained from *xz* scans of sixteen predetermined positions on the culture as previously described (1). airway surface liquid height was measured 5 min following the addition of the Rhodamine dextran and at designated time points over a period of 24 h in primary tracheal epithelial cultures from βENaC-Tg mice and WT littermates.

**Animals**

βENaC-Tg mice, backcrossed onto the C57Bl/6 background [1], were obtained from M. Mall (University of Heidelberg, Heidelberg, Germany). Male βENaC-Tg mice were mated with female C57Bl/6Cr1 wild-type (WT) mice (Charles River). All mice were bred in the animal facility of the Ghent University Hospital and maintained in standard conditions under a 12 h light-dark cycle and provided with a standard diet (Pavan, Brussels, Belgium) and chlorinated tap water *ad libitum*. The pups were genotyped before weaning and were weaned at day 21. All *in vivo* manipulations were approved by the local Ethics Committee for animal experimentation of the Faculty of Medicine and Health Sciences (Ghent University).

**Cigarette smoke exposure**

CS exposure started at an age of 7-8 weeks. In each experiment βENaC-Tg mice and their WT littermates were exposed whole body to cigarette smoke as described before [2]. In short, mice were exposed 5 days a week to the mainstream cigarette smoke of 5 cigarettes (Reference cigarette 3R4F without filter, University of Kentucky, Lexington, KY, USA), 4 times a day with a 30 minute smoke-free interval between exposures. A standard smoking apparatus was used with the smoking chamber adapted for a group a mice. A smoke/air ratio of 1/6 was obtained. The control mice were exposed to room air. The exposure period was either 4 or 8 weeks.

**βENaC immunohistochemistry**

Lung sections were evaluated for over-expression of the β-subunit of ENaC, using a rabbit polyclonal anti-βENaC antibody (5) at a dilution of 1:1,000. Unstained and hydrated paraffin sections were pretreated with 3% hydrogen peroxide, followed by antigen retrieval and incubation with a non-specific protein-blocking solution containing normal goat serum (Vector Laboratories, Inc., Burlingame, CA). Tissue sections were incubated overnight at 4°C with the primary antibody. As a secondary antibody, we used biotinylated goat anti-rabbit IgG. The immunoreactivity of βENaC was visualized using a peroxidase system (Vector Elite ABC Peroxidase Kit; Vector Laboratories, Inc.), followed by the use of a 3,3′-diaminobenzidine substrate kit (DAB-substrate kit; Vector Laboratories, Inc., Burlingame, CA) as previously described (6). Peroxidase reaction was stopped exactly after 2 min.

**RNA extraction and quantitative real-time polymerase chain reaction (qRT-PCR)**

The miRNeasy mini kit (Qiagen) was used to extract total lung RNA. cDNA was prepared with the Transcriptor Universal cDNA Master kit (Roche, Basel, Switserland) following manufacturer’s instructions. Target genes (Muc5ac, Muc5b, Cxcl1, Cxcl13, Ccl20) and reference genes Hprt-1 (Hypoxanthine phosphoribosyltransferase-1), Gadph (Glyceraldehyde-3-phosphate) and Tfcr (Transferrin receptor) were analyzed using TaqMan Gene Expression Assays (Applied Biosystems, Forster City, CA, USA). Serial dilutions of a mixture of all samples were included in each run and were used to obtain a standard curve. Reaction samples had a final volume of 20µl consisting of LightCycler 480 Probes Master (Roche), the specific primer/fluorogenic probe mix (Applied Biosystems) and 4µl of diluted cDNA. Identical amplification conditions were used for each of the target and reference genes. qRT-PCR reactions were setup in duplicate and were run on a Lightcycler 96® SW 1.1 instrument (Roche, Basel, Switserland). The amplification conditions consisted of a 10 minutes pre-incubation period at 95°C and 50 cycles of a two-step amplification (10 seconds at 95°C, 15 seconds at 60°C). Data were processed using the standard curve method. Expression of target genes was corrected by a normalization factor that was calcultated based on the expression of the 3 reference genes (Htpr-1, Gadph, Tfcr), using the geNorm applet according to the guidelines and theoretical framework preciously described (http://medgen.ugent.be/~jvdesomp/genorm/)[3].

**Goblet cell analysis**

Transversal sections were made from the paraffin-embedded left lung and were stained with Periodic acid-Schiff (PAS). PAS stains neutral mucins and acid mucins that contain significant amounts of sialic acid. Both major airway mucins (Muc5ac and Muc5b) are stained by PAS. Goblet cells were counted using Axiovision software (Zeiss) and were expressed as the number of goblet cells per millimetre basal membrane.

**Bronchoalveolar lavage (BAL)**

BAL was obtained 24 hours after the last smoke exposure, as described before [4]. Mice were weighed and sacrificed with an overdose of pentobarbital (Sanofi, Libourne, France). A tracheal cannula was placed and lungs were lavaged using 3 times 300µl HBSS, free of Ca^2+^ and Mg^2+^ and supplemented with 1% BSA, followed by 3 times 1 ml HBSS supplemented with 0.6mM EDTA. The lavage fractions were pooled and centrifuged and the cell pellet was resuspended in 200µl buffer (PBS with 1% BSA, 5mM EDTA and 0.1% Na^+^ azide). From this resuspended cell pellet, total cell counts were determined using a Bürker chamber and differential cell counts were performed on cytocentrifuged preparations after May-Grünwald-Giemsa staining. At least 400 cells were counted. Following the 8 week CS exposure, flow cytometric analyses of BAL cell were performed to obtain the number of macrophages, dendritic cells, neutrophils and CD4+ and CD8+ T-lymphocytes. The protein levels of Cxcl1 and Ccl20 in BAL fluid supernatant of mice were determined with an ELISA kit (R&D systems).

**Lung harvest and preparation of single-cell suspensions**

Following BAL, pulmonary and systemic circulation was rinsed with saline, supplemented with 5mM EDTA. The left lung was used for histology, as previously described [2, 5]. The major lobe of the right lung was harvested for the preparation of a single-cell suspension, as described previously [6]. In short, the lung was minced, digested, subjected to red blood cell lyses, passed through a 50µm cell strainer and kept on ice until labelling.

**Labelling of BAL cells and lung single-cell suspensions for flow cytometry**

First, the cells were incubated with FcR blocking antibody (anti-CD16/CD32, clone 2.4G2) to reduce nonspecific binding. Second, multicolor labelling reactions were performed to discriminate macrophages, dendritic cells, neutrophils and T lymphocytes. All reactions were performed on ice. Flow cytometric analyses of the 4 week CS exposure experiment were done using a FACSCalibur™ flow cytometer (BD Biosciences, San Diego, CA, USA). The analyses of the 8 week CS exposure experiment were performed on a BD LSRfortessa™ (BD Biosciences, San Diego, CA, USA). The macrophages and dendritic cells were discriminated using the methodology described by Vermaelen and Pauwels [7]. Macrophages were identified as CD11c^bright^ (APC-conjugated anti-CD11c; HL3, BD Biosciences) and high autofluorescent. Dendritic cells were distinguished as CD11c^bright^, low autofluorescent and MHC class II^high^ (PE-conjugated anti-I-A(b); AF6-120.1, BD Biosciences). T cell populations were labelled with and discriminated by FITC-conjugated anti-CD4 (GK1.5, BD Biosciences), FITC-conjugated anti-CD8 (53-6.7), APC-conjugated anti-CD3 (145-2C11) and PE-conjugated anti-CD69 (H1.2F3), a marker for activation of T lymphocytes. Due to the high amount of debris in BAL samples of βENaC-Tg mice, BAL samples were analyzed on the LSRFortessa™ and were alternatively labelled with Alexa Fluor 700 anti-CD45 (BD Biosciences), Pacific Blue anti-CD3 (Biolegend, San Diego, CA, USA), FITC-conjugated anti-CD4 (GK1.5), PE-conjugated anti-CD8 (BD Biosciences), APC-conjugated anti-CD11c (HL3) and APC-Cy7-conjugated anti-I-A(b) (Biolegend) to discriminate macrophages, dendritic cells and CD4^+^ en CD8^+^ lymphocytes. Neutrophils were discriminated using APC-conjugated anti-CD11c, biotin-labelled anti-CD11b with a secondary Steptavidin-PerCP labelling, FITC-conjugated Ly6C, PE-conjugated Ly6G and Alexa Fluor 700-conjugated anti-CD45 (BAL). These antibodies were all purchased from BD Biosciences. Data acquisition on the FACSCalibur™ was performed using CellQuest™ software and FACS DIVA™ software on the LSRFortessa™ (BD Biosciences). FlowJo Software (Tree Star Inc., Ashland, OR, USA) was used for data analysis.

**Quantification of lymphoid follicles**

In order to quantify lymphoid follicles, defined as dense accumulations of at least 50 lymphocytes, paraffin-embedded sections of the left lung were immunohistochemically stained with anti-CD3 (Dako) and anti-B220 (BD biosciences) [8]. The number of follicles was normalized for the total area of parenchyma that was scored.

**Quantification of emphysema**

In order to evaluate pulmonary emphysema, two complementary methods were used, the mean linear intercept (Lm) [9] and the destructive index (DI) [10]. The Lm is a measurement of alveolar space enlargement whereas the DI is a calculation of the percentage of destroyed alveolar walls. Both analyses were performed using the Image J software on hematoxylin and eosin (H&E) stained lung sections.

In short, for measuring the Lm, a 100 x 100 µm grid was placed on top of lung sections without artefacts, compression and hilar structures (bloodvessels or airways). Each intercept of alveolar wall on a line of the grid was counted and the total length of each line of the grid was divided by the total number of intercepts on that line. This results in an average distance between two alveolar walls, i.e. the Lm. For measurement of the DI a grid with 42 points was placed over the lung sections. For each point of the grid it was determined whether the structure underneath the point was normal (N) or destroyed (D). The DI is calculated as follows: DI = D/(D+N) x 100.

**Airway wall remodeling**

To evaluate the deposition of fibronectin and collagen, paraffin-embedded sections of the left lung was used for immunohistological staining, as described before [11]. Fibronectin was stained with mouse anti-fibronectin (Thermo-Scientific). Collagen was stained chemically with Sirius Red (Calbiochem, BadsSohen, Germany). The amount of collagen en fibronectin in the airway wall was quantified using the Axiovision software (Zeiss). The area of collagen or fibronectin was normalized to the length of the basement membrane (Pbm). All airways with a Pbm smaller than 2000 µm and cut in reasonable cross sections (defined by a ratio of maximal internal diameter greater than 0.5) were included.

**Lung function measurements**

Using the Flexivent System (SCIREQ, Montreal, Canada), baseline lung function was examined invasively in tracheostomized anaesthetized mice [12] after 4 weeks of CS exposure. The jugular vein was used to administer pancuronium bromide (1 mg/kg) (Inresa, Freiburg, Germany), which induces a neuromuscular blockade. The mice were ventilated with an average breathing frequency of 150 breaths/minute. Once the mice were stable, resistance (R) and dynamic compliance (C_dyn_) were measured using a ‘snapshot perturbation’ manoeuvre. The forced oscillation perturbation (Quick Prime 3) was applied to assess the tissue damping (G). Pressure-volume (PV) loops (PVs-V = PV-stepwise-volume regulated) were generated to measure the static compliance (C_stat_), total lung capacity (TLC) and hysteresis (area between inflating and deflating part of the PV loop). Both snapshot and quick prime 3 perturbations were performed 5 times and 3 PV loops were generated. An average was calculated of a minimum of 3 out of 5 measurements per perturbation with a coefficient of determination (COD) of at least 0.95.

**Statistical analyses**

Sigma Stat Software (SPSS 21.0, Chicago, IL, USA) was used to perform non-parametric tests (Kruskall-Wallis and Mann-Whitney-U). Reported values are expressed as mean ± SEM. P-values < 0.05 were considered to be significant.

**References**

1. Johannesson B, Hirtz S, Schatterny J, Schultz C and Mall MA CFTR regulates early pathogenesis of chronic obstructive lung disease in betaENaC-overexpressing mice. PloS one. 2012; 7: e44059.

2. Bracke KR, D'Hulst A I, Maes T, Moerloose KB, Demedts IK, Lebecque S*, et al.* Cigarette smoke-induced pulmonary inflammation and emphysema are attenuated in CCR6-deficient mice. J Immunol. 2006; 177: 4350-4359.

3. Vandesompele J, De Preter K, Pattyn F, Poppe B, Van Roy N, De Paepe A*, et al.* Accurate normalization of real-time quantitative RT-PCR data by geometric averaging of multiple internal control genes. Genome biology. 2002; 3: RESEARCH0034.

4. Bracke KR, Verhamme FM, Seys LJ, Bantsimba-Malanda C, Cunoosamy DM, Herbst R*, et al.* Role of CXCL13 in cigarette smoke-induced lymphoid follicle formation and chronic obstructive pulmonary disease. Am J Respir Crit Care Med. 2013; 188: 343-355.

5. Bracke KR, D'Hulst A I, Maes T, Demedts IK, Moerloose KB, Kuziel WA*, et al.* Cigarette smoke-induced pulmonary inflammation, but not airway remodelling, is attenuated in chemokine receptor 5-deficient mice. Clin Exp Allergy. 2007; 37: 1467-1479.

6. Vermaelen KY, Carro-Muino I, Lambrecht BN and Pauwels RA Specific migratory dendritic cells rapidly transport antigen from the airways to the thoracic lymph nodes. J Exp Med. 2001; 193: 51-60.

7. Vermaelen K and Pauwels R Accurate and simple discrimination of mouse pulmonary dendritic cell and macrophage populations by flow cytometry: methodology and new insights. Cytometry A. 2004; 61: 170-177.

8. Demoor T, Bracke KR, Maes T, Vandooren B, Elewaut D, Pilette C*, et al.* Role of lymphotoxin-alpha in cigarette smoke-induced inflammation and lymphoid neogenesis. Eur Respir J. 2009; 34: 405-416.

9. Thurlbeck WM Measurement of pulmonary emphysema. Am Rev Respir Dis. 1967; 95: 752-764.

10. Saetta M, Shiner RJ, Angus GE, Kim WD, Wang NS, King M*, et al.* Destructive index: a measurement of lung parenchymal destruction in smokers. Am Rev Respir Dis. 1985; 131: 764-769.

11. Palmans E, Kips JC and Pauwels RA Prolonged allergen exposure induces structural airway changes in sensitized rats. Am J Respir Crit Care Med. 2000; 161: 627-635.

12. Lanckacker EA, Tournoy KG, Hammad H, Holtappels G, Lambrecht BN, Joos GF*, et al.* Short cigarette smoke exposure facilitates sensitisation and asthma development in mice. Eur Respir J. 2013; 41: 1189-1199.

**Supplementary figures**

**S1 Fig. Goblet cell metaplasia and mucin expression upon 8 weeks of air or CS-exposure.**

**(A)** Goblet cell count. n = 8-11/group. **(B)** mRNA expression of Muc5ac. **(C)** mRNA expression of Muc5b. Expression data normalized for 3 household genes (Hprt1, Gapdh, and Tfrc). n = 8/group.

*p<0.05, **p<0.01, ***p<0.001

**S2 Fig. CS-induced inflammation in BAL upon 8 weeks of CS exposure.**

**(A)** Total inflammatory cell count in BAL. **(B)** Number of macrophages in BAL. **(C)** Number of neutrophils in BAL. **(D)** Number of lymphocytes in BAL. n = 8-11/group.

*p<0.05, **p<0.01, ***p<0.001

**S3 Fig. Cigarette smoke-induced alveolar destruction is increased in βENaC-Tg mice after 8 weeks of air or CS exposure**

**(A)** Mean linear intercept (Lm) after 8 weeks of air or CS exposure. **(B)** Destructive index (DI) after 8 weeks of air of CS exposure. n = 8-11/group.

**S4 Fig. 8 weeks of cigarette smoke exposure does not induce airway wall remodelling in WT and βENaC-Tg mice.**

**(A)** Deposition of fibronectin in the airway wall. Normalized for perimeter basement membrane. **(B)** Deposition of collagen in the airway wall. Normalized for perimeter basement membrane. n = 8-11/group.
